# Atypical birdsong and artificial languages provide insights into how communication systems are shaped by learning, use, and transmission

**DOI:** 10.3758/s13423-016-1107-5

**Published:** 2016-07-20

**Authors:** Olga Fehér

**Affiliations:** School of Philosophy, Psychology and Language Sciences, University of Edinburgh, Edinburgh, UK

**Keywords:** Birdsong, Artificial languages, Language evolution

## Abstract

In this article, I argue that a comparative approach focusing on the cognitive capacities and behavioral mechanisms that underlie vocal learning in songbirds and humans can provide valuable insights into the evolutionary origins of language. The experimental approaches I discuss use abnormal song and atypical linguistic input to study the processes of individual learning, social interaction, and cultural transmission. Atypical input places increased learning and communicative pressure on learners, so exploring how they respond to this type of input provides a particularly clear picture of the biases and constraints at work during learning and use. Furthermore, simulating the cultural transmission of these unnatural communication systems in the laboratory informs us about how learning and social biases influence the structure of communication systems in the long run. Findings based on these methods suggest fundamental similarities in the basic social–cognitive mechanisms underlying vocal learning in birds and humans, and continuing research promises insights into the uniquely human mechanisms and into how human cognition and social behavior interact, and ultimately impact on the evolution of language.

The structural complexity and nearly infinite expressive flexibility of human language make it unique among the communication systems of the animal kingdom. Language has enabled humans to efficiently learn, innovate, and collaborate-- abilities that form the foundations of human culture today. There is no doubting the importance of linguistic communication in human ecological success, but we know very little about how the human cognitive system evolved to allow language to emerge. In this article, I discuss a comparative behavioral approach that focuses on the social, communicative, and learning mechanisms underlying birdsong and language and that aims to identify which of these may be general characteristics of vocal learning and which may be specific to language and human cognition. A more thorough knowledge of these mechanisms should inform us about how language, through a dynamic relationship with the human cognitive system, could have evolved the complex structure and expressive power that it possesses.

No closely related organism possesses a vocal communication system that could serve as a biological model for language evolution, and no easily observable communication systems bear obvious similarities to language that could serve as a behavioral model. For instance, our closest relatives appear to have a communication system designed along entirely different principles, and it is unclear whether apes show any vocal learning at all. There is some evidence for the flexible vocal behavior of chimps (Watson et al., 2015), but this seems to involve only contextual learning—the use of existing signals in novel contexts (Fischer, Wheeler, & Higham, 2015)—rather than vocal production learning, which involves the modification of the signals themselves. Another strand of research focuses on other socially learned, albeit noncommunicative, behaviors in apes, such as tool use, that may be similar to language in being hierarchically organized and culturally transmitted (Gruber, Muller, Strimling, Wrangham, & Zuberbühler, 2009).

A different approach involves looking at the convergent evolution of some of the capacities underlying language (Fitch, 2010), and a consensus is emerging that comparative methods based on this approach can yield valuable insights into aspects of human linguistic communication (Berwick, Beckers, Okanoya, & Bolhuis, 2012; Bolhuis & Everaert, 2013; Doupe & Kuhl, 1999). Vocal production learning (Janik & Slater, 2000), though rare, is present in other animals. Vocal learners are defined by their ability to modify their vocalizations on the basis of auditory input; this is not the same as other forms of auditory learning, such as the ability of dogs or apes to learn associations between sounds and meanings, which are examples of comprehension learning. Vocal learning evolved several times independently in distantly related lineages (Jarvis, 2004), and it may serve quite distinct roles in different animal taxa, such as individual identification and group cohesion in dolphins and parrots (Berg, Delgado, Okawa, Beissinger, & Bradbury, 2011; Janik & Slater, 1998) and mate attraction and territory defense in songbirds and whales (Searcy & Andersson, 1986; Tyack, 1981). Despite the ecological and perhaps functional dissimilarities, many behavioral traits are common to all vocal learners, including humans: Vocal learners acquire species-typical vocal behavior during an early sensitive period by imitating adult individuals (Marler, 1970); they preferentially imitate sounds of their own species (Thorpe, 1958); they require auditory feedback to progress through normal vocal development (Konishi, 1964); the absence of exposure to adults leads them to produce abnormal vocalizations (Fromkin, Krashen, Curtiss, Rigler, & Rigler, 1974; Immelmann, 1969); and they learn their communication systems through social learning, so that social interactions affect the outcome of the learning process (Baptista & Gaunt, 1997; Kuhl, Tsao, & Liu, 2003).

Vocal learning has been most extensively studied in songbirds: By now, we know that birds and humans share not only many of the behavioral mechanisms underpinning vocal learning (Doupe & Kuhl, 1999), but also some of the same genetic (Haesler et al., 2004), neural (Jarvis, 2004), and peripheral (Elemans et al., 2015) mechanisms. The approach I highlight here relies on studying learners’ responses to atypical input—that is, song or linguistic input containing modified or atypical versions of features that are widespread in wild-type song and natural language. This approach is useful for investigating several aspects of song and language learning. First, it reveals the biases and limitations that learners show while acquiring and reproducing atypical input. These constraints can be studied in adults or juveniles by using experiments that involve short episodes of rapid acquisition, but can also be studied over long, developmental timescales by collecting acoustic data from birds and children during their ontogeny. Second, by observing how learners use abnormal songs and languages in social groups, we can investigate the interactive mechanisms that shape vocal communication systems. Finally, studying how these abnormal songs and languages are transmitted and acquired by successive generations of learners gives us clues to how cultural evolutionary processes shape these communication systems in the wild. I will highlight some insights we have gained from studying these processes using atypical song or linguistic input in songbirds and humans.

## Learning an atypical system

### Atypical linguistic variation

Variation is widespread in language and is present at all levels of structural organization: in individuals, within populations, and between populations. For example, languages exhibit variation in sentence structure (e.g., *Annie baked the cake* and *the cake was baked by Annie* are paraphrases), in morphology (e.g., the plural in English can be realized as [s], in *cats*, or [z], in *dogs*, or [ɪz], in *horses*), and in the lexical items available to express a given concept (e.g., the synonyms *mouth*, *oral cavity*, *trap*, *gob*, and *piehole*). Crucially, linguistic variation tends not to be random or fully unpredictable (Givon, 1985; Sossinka & Bohner, 1980; Trillo & Vehrencamp, 2005); rather, the use of variants is conditioned on grammatical or social context. For instance, one would not expect to hear the words *oral cavity* and *gob* in identical situations. Indeed, variation is an essential aspect of social communication: Social context is a reliable predictor of the choice of linguistic variants (Labov, 1966), and, in turn, the variants carry socially relevant information to speakers (Meyerhoff, 2008). The facts that variation is meaningful rather than random or unpredictable and that this appears to be a universal feature of natural languages suggest that people possess cognitive biases that constrain language in this way.

Psycholinguists have used artificial-language paradigms exhibiting unpredictable variation to study how learners acquire and reproduce atypical linguistic input, in an attempt to uncover these biases. In these paradigms, people are trained on a language featuring objects or actions described by arbitrary words, with more than one competing linguistic variant for each meaning, presented in different proportions. After training, participants are tested on the language by having to reproduce it. A basic finding of these studies is that children tend to *regularize*—that is, to eliminate unpredictable variation by dropping the competing variants and using only one per meaning. Adults, on the other hand, have no problem using multiple linguistic variants to describe the same meaning. Moreover, adults tend to produce the variants in the same proportion that was present in their input, effectively probability-matching the variants in their training language (Hudson Kam & Newport, 2005; Wonnacott, 2011). The contrast between child and adult learners may not be so sharp, however: Subsequent studies have shown that adults also regularize under certain conditions (Hudson Kam & Newport, 2009; Wonnacott, Newport, & Tanenhaus, 2008). Other types of atypical languages, including atypical word order patterns (Culbertson, Smolensky, & Legendre, 2012) and atypical case-marking systems (Fedzechkina, Jaeger, & Newport, 2012), have also been used to demonstrate that learners alter unnatural input languages, changing them to more closely resemble natural languages.

### Isolate song

Similarly to humans, songbirds exhibit biased imitation when they are trained on vocal input containing atypical features. A useful model for such abnormal vocal input is provided by untutored or isolate song. *Isolate song* is the improvised song of young birds who grow up in acoustic isolation, and has characteristics that differentiate it from normal, wild-type song. For instance, in zebra finches, isolate song tends to be more variable, both phonetically and at the level of the song bout, and it contains syllables that are often repeated and longer in duration than those seen in wild-type song (Immelmann, 1969; Williams, Kilander, & Sotanski, 1993). Although juvenile zebra finches are capable of imitating adult song extremely accurately, when they are trained by adult birds who sing isolate song, they tend to modify the abnormal acoustic features and produce songs that are more similar to normal, wild-type song (Fehér, Wang, Saar, Mitra, & Tchernichovski, 2009). The pupils of isolate adults are also sensitive to the naturalness of the statistical distributions in their input: The number of syllable repetitions in the tutor song is faithfully copied when it is within the normal range, but it is decreased when it exceeds the wild-type range (Fehér et al., 2009).

These experimental findings documenting how learners respond to atypical input suggest that humans and songbirds come to the learning process with at least some implicit expectations about the likely properties of the system they will be learning. In songbirds, these *innate biases* can appear in the form of preferential imitation of certain song types (Nowicki, Peters, Searcy, & Clayton, 1999; ter Haar, Kaemper, Stam, Levelt, & ten Cate, 2014) or an ability to “fill in the gaps”, i.e. produce species-typical songs even in the absence of complete, wild-type song models (Rose et al., 2004; Soha & Marler, 2001). However, *perceptual* or *learning biases* may not be strong and domain-specific—rather, they might involve limitations due to physiology, memory, or perception. Regardless of their precise nature, they must play a role in shaping the structure of socially learned communication systems (Christiansen & Chater, 2008). Therefore, studying how individuals react to features that are not normally characteristic of their communication systems can teach us not only about the relationship between cognitive systems and the structure of the communication system they have produced, but also how these communication systems might have come about.

## Using an atypical communication system

Language and birdsong are used in interaction between individuals and are socially learned in a rich and complex communicative environment. Young birds and children learn to vocalize amidst a web of complex social interactions with multiple individuals: their parents, other related and unrelated adults, and their siblings as well as unrelated peers. Findings from several decades of research into the social mechanisms that influence vocal learning have suggested that shared mechanisms underlie vocal learning in birds and humans (Fitch, Huber, & Bugnyar, 2010). The communicative role of sending and receiving messages is central to both language and birdsong, so it is reasonable to expect that interaction, in addition to learning, should play a role in shaping their structural properties. In the next section, I will discuss some interactive processes that seem to be a characteristic of vocal communication systems in general, and I will highlight evidence from birdsong and language to suggest that these processes likely have important evolutionary implications. I argue that atypical song and linguistic input offer a great opportunity to systematically investigate how social mechanisms may have led to the current structural properties of birdsong and language.

### Communicative mechanisms in human language

During communicative interaction, human interlocutors modify their behavior to match that of their partners, in a process called *convergence* or *alignment* (Pickering & Garrod, 2004). The process of linguistic convergence has been demonstrated in natural dialogue (e.g., Levelt & Kelter, 1982; Schenkein, 1980) and in the laboratory (Branigan, Pickering, & Cleland, 2000) at all levels of linguistic structure. As a mechanistic explanation for linguistic convergence, Pickering and Garrod (2004) appealed to *priming*, people’s tendency to reuse a linguistic form that has just been used by their communication partner. Although low-level linguistic alignment may be largely due to automatic processes, the degree of alignment seems to be socially mediated (Weatherholtz, Campbell-Kibler, & Jaeger, 2014). Convergence may play an important role in communication by simplifying and accelerating comprehension and production, and it may also serve a social function by reinforcing cooperation. However, the opposite is also observed: People signal social distinctiveness and reinforce social identity by diverging linguistically from their conversation partner (Bourhis, Giles, Leyens, & Tajfel, 1979), which promotes cultural diversification. Additional communicative aspects of language, such as turn-taking, which may at first glance appear culturally quite distinct, also seem to be governed by universal communicative principles: Stivers et al. (2009) showed that the mechanistic processes driving conversational turn-taking, such as minimizing overlap and silent intervals between turns, are shared across languages from extremely different cultures. As I will discuss in the next section, similar interactive mechanisms serving similar social goals can be observed in birdsong.

### Interactive processes in birdsong

Vocal convergence in wild songbirds is abundant: For instance, European siskin pairs imitate each other’s flight calls (Mundinger, 1970), and song sparrows match their song types (Beecher, Campbell, Burt, Hill, & Nordby, 2000) and their song repertoires (Beecher, Stoddard, Campbell, & Horning, 1996) with those of neighboring birds. Moreover, song sparrows exhibit different levels of song-type matching, depending on the identity of the singing partner (Stoddard, Beecher, Campbell, & Horning, 1992), and they refrain from singing shared songs when a stranger’s song is played from the neighbor’s territory (Beecher et al., 1996). Dueting, a particular type of song matching that requires high temporal precision in alternating the song elements, seems to signal cooperation between pairs to strengthen pair bonding (Hall, 2004; Templeton, Ríos-Chelén, Quirós-Guerrero, Mann, & Slater, 2013). Convergence is also not restricted to songbirds: Other birds capable of vocal learning, such as cockatoos (Scarl & Bradbury, 2009) and wild parrots (Bradbury, 2004), exhibit it as well. In the lab, social call convergence has also been demonstrated in budgerigars (Farabaugh, Linzenbold, & Dooling, 1994). Interestingly, in parallel with humans, overlap avoidance has also been demonstrated in songbirds—for example, in white-throated sparrows (Wasserman, 1977), Eastern meadowlarks (Knapton, 1987), and nightingales (Hultsch & Todt, 1982). Moreover, overlapping singing is perceived as an aggressive signal (Brindley, 1991; Dabelsteen, Gregor, Holland, Tobias, & Pedersen, 1997; Todt & Naguib, 2000; Wolffgramm & Todt, 2016), and overlapping singers are judged to be a greater threat than nonoverlappers (Naguib & Todt, 1997).

Convergence occurs at developmental timescales as well, when young birds that are housed together during song ontogeny produce similar songs as adults. This process may involve quite different social-learning mechanisms and is arguably functionally distinct from adult song or call convergence; however, it may also have a large impact on the maintenance of song culture. Developmental convergence has been demonstrated in laboratory studies: Although zebra finch siblings tutored by an adult in a normal social setting often diverge from each other (Tchernichovski & Nottebohm, 1998), when they are raised in peer groups without an adult tutor, they converge on shared songs, and their final songs are as similar to each other as those of birds raised by a single tutor (Jones, ten Cate, & Slater, 1996; Volman & Khanna, 1995). Interestingly, when young birds are raised in this type of group isolation, without access to adult song models, the songs they produce are not only similar to each other, but often more like wild-type song and less like isolate songs than those that develop in isolated individuals (Chaiken, Gentner, & Hulse, 1997). This suggests that interaction provides a mechanism to amplify the birds’ biases for normal song. Moreover, when young zebra finches are trained on their own developing song (in experiments that involve playing back an isolate bird’s song to itself after a short delay throughout song ontogeny), wild-type song features emerge without real social interaction or external song input (Fehér, Ljubičić, Suzuki, Okanoya, & Tchernichovski, 2016). The moment-to-moment processes underlying this developmental emergence of normal song features are unknown, but Benichov et al. (2015) recently developed a technique that now makes it possible to investigate this in a controlled manner. The authors used a vocal robot that produced vocalizations according to a predetermined pattern, and they demonstrated that both female zebra finches (who do not sing) and males synchronize their social calls. The advantage of this method is that it gives experimenters full control over the interactive vocal input.

### Interactive mechanisms impact on the cultural evolution of language

Social mechanisms determine the ways in which vocal communication systems are used and transmitted, and they probably played a central role in the emergence of these systems. According to an influential account, pressures for information sharing to avoid costly trial-and-error learning resulted in shared linguistic conventions among members of a social group, the closest being family groups (Fitch, 2004). Linguistic conventions may have evolved through convergence and divergence, which would have eventually led to shared linguistic markers and the emergence of distinct dialects. This, in turn, would have strengthened the cooperation between individuals who spoke the same dialect and made it easier to identify “outsiders” who spoke a different dialect (Nettle & Dunbar, 1997). The cultural evolutionary consequences of this would be linguistic diversity on a large geographical scale and shared use on a small scale, which is what we observe in nature.

The interactive mechanisms involved in language use may directly impact on linguistic structure, and artificial languages provide an opportunity to study this relationship. In recent work, we extended the paradigms commonly used to study individual learning biases (discussed above) and tested whether communicative interaction would lead to the elimination of unpredictable linguistic variation, following the intuition that convergence could also contribute to the shaping of language structure by acting as a mechanism to amplify individual biases through interaction. We taught participants artificial languages that exhibited unpredictable variation in singular marking (i.e., we trained pairs of participants on artificial languages that differed in the proportion of training trials on which the singular was marked by a nonce word) and allowed the participants to communicate using these languages. We found that the interlocutors not only converged on a shared system of using the singular marker, but interaction also led to the elimination of unpredictable variation (Fehér, Ritt, & Smith, 2016; Smith, Fehér, & Ritt, 2014). To directly test the link between priming and regularization (i.e., the elimination of unpredictable variation), we ran a series of experiments using artificial languages with unpredictable syntactic variation in a variety of interactive situations (Fehér, Wonnacott, & Smith, in press). We found evidence for syntactic priming in three different communicative contexts: in human–human interaction, human–computer interaction, and a human–computer interaction condition in which people were led to believe that they were interacting with a human. Although priming was present in all conditions, regularization was much greater when people were interacting with another human or believed they were doing so. These findings suggest that priming is a low-level, largely automatic process, and that *reciprocal* priming in human dyadic interaction, together with communicative intentions, may directly shape the structural properties of linguistic systems.

To investigate the role of social information further and see whether the presence of multiple speakers (in an individual-learning, noninteractive setting) would influence regularization, we recently showed that when people learn languages with unpredictable lexical variation from multiple speakers, they reproduce the variability in their input language, but only if their teachers were individually variable themselves. If the speakers were individually consistent but differed in their uses of words (yielding population-level variability), participants imitated the majority variant (Fehér, Kirby, & Smith, 2014). This shows that learners are sensitive to minimal social cues in their input when acquiring artificial languages, and that the tendency of learners to regularize unpredictable variation is influenced by these cues. Another recent study using a similar input language showed that minimal social cues are sufficient to induce divergence between groups of interlocutors (Kerr & Smith, 2016). This experimental method, based on the minimal-group paradigm (Tajfel, Billig, Bundy, & Flament, 1971), offers further opportunities to explore the effect of the social structure on how languages are shaped by their use in social groups.

### Song culture is shaped by social processes

Social-learning strategies that may influence vocal learning include directed social learning (Coussi-Korbel & Fragaszy, 1995) or model-based imitation (i.e., a tendency to imitate influential individuals) and also conformity (i.e., the tendency to imitate majority behavior; Richerson & Boyd, 2005). Social-learning strategies play an important role in cultural evolution, as they allow social animals to quickly acquire adaptive behaviors without trial-and-error learning (Galef & Laland, 2005). Their exact role in vocal learning is not clear. European siskins were found to preferentially imitate the songs of high-ranking males (Mundinger, 1970), and white-crowned sparrows, at the time of establishing territories, selectively retain songs that most closely match those of their neighbors (Nelson & Marler, 1994). Chipping sparrows do something similar, but in addition to selective retention, they alter their own song to even more closely match their neighbor’s (Liu & Nottebohm, 2007). These processes contribute to the maintenance of cultured phenotypes and, in the long term, to the stability of vocal culture, which may promote adaptation to local environments driven by female preferences (Nottebohm, 1972). Therefore, local dialects may directly increase biological fitness, and in this case, social learning should serve the goal of maintaining a stable local dialect. Alternatively, local dialects may serve social functions such as promoting group cohesion. A particularly interesting case is the colony song of the Panamanian yellow-rumped cacique, *Cacicus c. cela*. These birds, like many other songbirds, sing colony-specific songs, but in contrast to the generally stable song dialects of most geographically distributed populations, cacique songs can change and diffuse very rapidly. Males and females do not form pair bonds and males are not territorial, but they use the colony song to attack intruders in groups. The song therefore seems to serve a group identification function, acting as some sort of password (Feekes, 1982). This strong convergence and rapid turnover is similar to what occurs in humpback whale song cultures, in which individuals entering a population may introduce new songs that are rapidly adopted by the entire population (Noad, Cato, Bryden, Jenner, & Jenner, 2000).

Song dialects clearly serve both ecological and social functions that are weighed differently in different species. Laboratory studies using isolate song suggest an intricate relationship between the social and ecological aspects of song: Although zebra finch males singing isolate song were shown to have inferior reproductive success, isolate song was readily accepted, and in some cases preferred, as a valid song model by juveniles (Williams et al., 1993). On the other hand, male cowbirds do not imitate the songs that females prefer the most (West & King, 1986), and females show a stronger preference for untutored songs, which incidentally provoke an aggressive response from normally raised males (West & King, 1980). These findings suggest complex and species-specific social mechanisms. Methods involving virtual social environments, where juvenile birds are exposed to videos of interacting and singing birds (Ljubičić, Hyland Bruno, & Tchernichovski, 2016), now allow for the investigation of these mechanisms under full experimental control.

## Transmitting an atypical communication system

The communication systems of vocal learners are culturally transmitted via social learning. Cultural transmission can lead to rapid changes in the structure of a vocal communication system, because once the biological foundations for social and vocal learning are in place, the organism does not need to undergo further biological evolution for the system to adapt to its user and change according to its user’s needs and behavior. Vocal learners are in a dynamic relationship with their communication systems, whose features reflect their cognitive biases and predispositions (Christiansen & Chater, 2008; Kirby, 1999, 2016). The fingerprints of these cognitive biases are observable in the universally shared properties of natural languages or songs. Linguistic typology is devoted to the description of such universal properties, but it gives us only a momentary picture of the constraints on cross-linguistic variation, without showing the mechanisms through which they appeared. Luckily, cultural evolutionary processes can be studied in the laboratory via iterated-learning paradigms in which the behavioral outputs of one individual or a group of individuals serve as input to the next generation of learners (Kirby, Cornish, & Smith, 2008; Smith, Kirby, & Brighton, 2003). Iterated-learning experiments allow the experimenter to manipulate the pressures acting on languages as they are learned and transmitted and to study how these pressures shape the evolving linguistic systems (Kirby, Tamariz, Cornish, & Smith, 2015; Reali & Griffiths, 2009). In a demonstration of how individual biases and transmission interact, Smith and Wonnacott (2010) taught adult learners a language with unpredictable variation and found that, whereas individual learners reproduced the variation by using the variants in roughly the same proportions that had been present in their input language (as adults have tended to do in other studies mentioned above), their weak regularization biases accumulated across multiple generations and gradually led to predictable languages.

Birdsong also evolves culturally and exhibits both geographical and dialectal variation (Marler, 1960), and the evolution of this variation in the wild has been under intense investigation (for reviews, see Podos & Warren, 2007; Riebel, Lachlan, & Slater, 2015). Song evolution can also be studied in the laboratory by using iterated learning. When isolate song is transmitted across multiple generations, wild-type song features emerge (Fehér et al., 2009). This process is very quick: Within four or five learning generations, the songs become indistinguishable from normal zebra finch songs. Moreover, although the social environment affects what song features normalize more rapidly, the changes occur in both impoverished and rich social environments. In a colony founded by an isolate male (i.e., a rich social environment), females and siblings provide not only social feedback, but also call syllables for the young males to imitate. This makes the evolving songs richer in terms of the diversity of song elements, resulting in the fast evolution of song rhythm. In an impoverished social context, when the young birds are tutored one-on-one by an isolate adult, they receive more individual attention, and perhaps more intense acoustic feedback. In this setting, the internal spectral structure of the songs evolves more quickly. Exactly how social variables influence cultural evolutionary processes in birdsong and language is a fascinating question and one that is ripe for future investigation.

## Conclusions

Using atypical input has allowed us to investigate the effects of individual learning, interaction, and transmission on the cultural evolution of birdsong and language. We are learning more and more about the independent and combined contributions of all these processes to how communication systems change and evolve. Investigation using atypical languages and songs continues in several directions. One particularly exciting area is the study of social and interactive influences on vocal learning. The presence of so many parallels between the social processes involved in vocal learning in birds and humans suggests fundamental similarities in the basic social–cognitive mechanisms underlying this ability. Continuing research should highlight the specific differences between birds and humans, and this in turn would yield insights into the unique features of the human cognitive system and human social interactions that resulted in the evolution of language as a communicative tool with unparalleled expressive power and flexibility.
